# Risk factors of non-diagnostic percutaneous liver tumor biopsy: a single-center retrospective analysis of 938 biopsies based on cause of error

**DOI:** 10.1007/s11604-024-01703-3

**Published:** 2024-11-14

**Authors:** Shintaro Kimura, Miyuki Sone, Shunsuke Sugawara, Chihiro Itou, Takumi Oshima, Mizuki Ozawa, Rakuhei Nakama, Sho Murakami, Yoshiyuki Matsui, Yasuaki Arai, Masahiko Kusumoto

**Affiliations:** 1 Department of Diagnostic Radiology, National Cancer Center Hospital, 5-1-1 Tsukiji, Chuo-ku, Tokyo, 104-0045 Japan; 2 Cancer Medicine, The Jikei University Graduate School of Medicine, Tokyo, Japan; 3 Department of Urology, National Cancer Center Hospital, Tokyo, Japan

**Keywords:** Liver tumor biopsy, Non-diagnostic result, Risk factor

## Abstract

**Purpose:**

To evaluate the risk factors of non-diagnostic results based on cause of error in liver tumor biopsy.

**Materials and methods:**

This single-institution, retrospective study included 843 patients [445 men, 398 women; median age, 67 years] who underwent a total of 938 liver tumor biopsies between April 2018 and September 2022. An 18-G cutting biopsy needle with a 17-G introducer needle was used. Ultrasound was used as the first choice for image guidance, and computed tomography was alternatively or complementarily used only for tumors with poor ultrasound visibility. Non-diagnostic biopsies were divided into two groups depending on the cause of error, either technical or targeting error. Biopsies in which the biopsy needle did not hit the target tumor were classified as technical error. Biopsies in which insufficient tissue was obtained due to necrosis or degeneration despite the biopsy needle hitting the target tumor were classified as targeting error. This classification was based on pre-procedural enhanced-imaging, intro-procedural imaging, and pathological findings. Statistical analysis was performed using binary logistic regression.

**Results:**

The non-diagnostic rate was 4.6%. Twenty-six and seventeen biopsies were classified as technical and targeting errors, respectively. In the technical error group, tumor size ≤ 17 mm and computed tomography-assisted biopsy due to poor ultrasound visibility were identified as risk factors (p < 0.001 and p = 0.021, respectively), and the tumors with both factors had a significantly high risk of technical error compared to those without both factors (non-diagnostic rate: 17.2 vs 1.1%, p < 0.001). In the targeting error group, tumor size ≥ 42 mm was identified as a risk factor (p = 0.003).

**Conclusion:**

Tumor size ≤ 17 mm and computed tomography-assisted biopsy due to poor ultrasound visibility were risk factors for technical error, and tumor size ≥ 42 mm was a risk factor for targeting error in liver tumor biopsies.

## Introduction

Percutaneous needle biopsy performed under imaging guidance is a safe procedure with high diagnostic accuracy, cementing its widespread application for tissue sampling from various organs [1–10]. In liver tumor biopsy, recent studies report a diagnostic accuracy of 93–96% [11, 12]. However, it is sometimes challenging to collect sufficient tissue from small tumors, cystic tumors, and large tumors with severe degeneration, which may be the target of diagnostic percutaneous needle biopsy or genomic molecular analysis [13–16]. Although diagnostic failure is rare, it presents a crucial problem because non-diagnostic results may lead to an incorrect treatment strategy and require the patient to undergo re-biopsy or surgery, which is burdensome for the patient, culminating in delays in the initiation of appropriate treatment. Several factors, including tumor size, biopsy technique (core needle biopsy or fine needle aspiration), number of needle passes, use of contrast media for ultrasound, and rapid on-site cytologic evaluation, may have been implicated in the diagnostic accuracy of percutaneous liver tumor biopsy (PLTB) [17–21]. However, the results of these studies have not necessarily been consistent, and the risk factors influencing non-diagnostic results remain unclear. Previous studies treated non-diagnostic results as one group, which included various causes of error, and therefore may have been unable to identify the specific clinical factors affecting the non-diagnostic result. We consider that there are two reasons for non-diagnostic biopsies: failure to puncture the target tumor and insufficient tissue sampling due to the tumor heterogeneity, and the analysis based on the cause of errors could be effective to identify the clinically relevant factors associated with non-diagnostic result in PLTB.

The purpose of this study was to determine the factors associated with non-diagnostic results based on the cause of error in PLTB using the coaxial technique.

## Materials and methods

### Study design

This was a single-center, retrospective observational study. The study was approved by the Institutional Review Board of our institution (2023-262) and conformed to the ethical guidelines of the 1975 Declaration of Helsinki. Informed consent was obtained by giving patients the option to optout by public announcement, and the need for written informed consent was waived owing to the retrospective design of the study, which entailed only a review of medical records.

### Study cohort

**Fig. 1:**
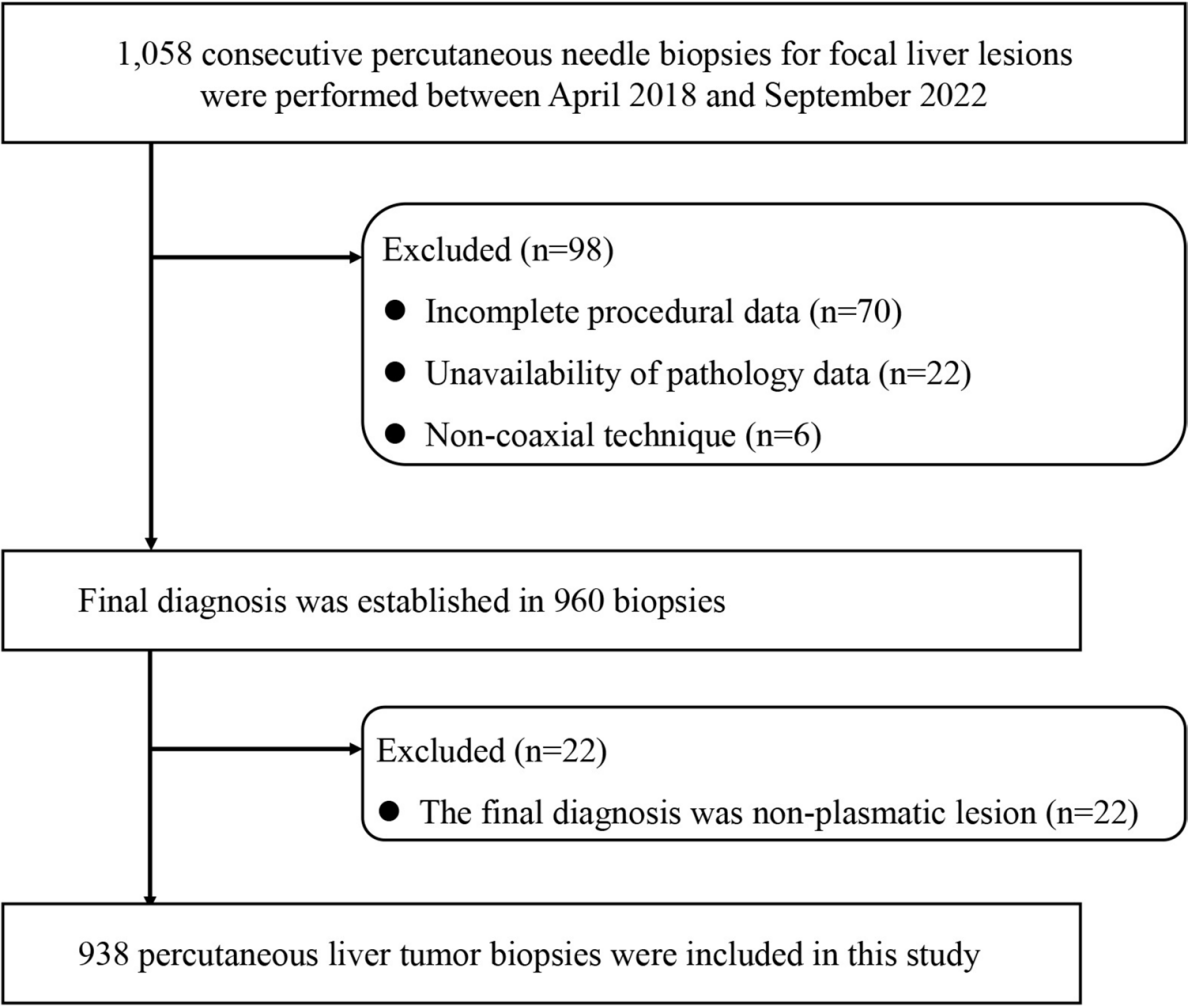
Flowchart of patient selection

The flow chart for study enrollment is shown in Fig. 1. A total of 1,058 consecutive percutaneous needle biopsies for focal liver lesions were performed between April 2018 and September 2022. Patients were considered ineligible if their clinical data, procedural details, or pathological data were incomplete or the biopsy was performed using a non-coaxial technique. As a result, 98 PLTBs were excluded, and the remaining 960 PLTBs were reviewed for final diagnosis. Twenty-two biopsies were finally diagnosed as non-neoplastic lesions and were excluded from this study. As a result, 938 PLTBs for 843 patients were included in the analysis of the risk factor for non-diagnostic results (Fig. 1).

### Biopsy procedure

PLTB was performed by nine interventional radiologists with an average of 12 (3–26) years’ interventional radiology experience. All procedures were performed at the interventional radiology suite with an angio-computed tomography (CT) system (INFX-8000C/Aquillion 16; Canon Medical Systems, Tochigi, Japan) and ultrasound (US) (Aplio i800, Aplio 300 or Nemio XG; Canon Medical Systems, Tochigi, Japan) under local anesthesia. The initial imaging guidance modality was US; however, when the target tumor was not clearly visualized on US due to the echogenicity or location such as near hepatic dome, CT were performed complementarily or alternatively. In CT-guided biopsy, intermittent CT-fluoroscopic guidance consisting of 3 slices of 4-mm thickness was used without iodinated contrast media. An 18-G cutting biopsy needle (MISSION; Becton Dickinson, NJ, USA, BARD MAGNUM; Becton Dickinson, or Pro-Mag; Argon Medical Devices, TX, USA) and a 17-G introducer needle were used for the procedures. For heterogeneous tumors, the viable area as assessed by pre-procedural enhanced CT or magnetic resonance imaging (MRI) was targeted for PLTB.

### Data collection

The patient-related demographic characteristics, tumor-related data, procedure-related data, major complications, and pathological results were retrospectively collated from the patients’ electronic medical records. The patients’ demographic factors, including age, sex, body mass index, as well as fatty liver, and ascites were documented. The tumor-related data encompassed the maximum diameter, depth, and location of the lesion. The maximum diameter of the target lesion was measured on preprocedural axial CT or MRI. The tumor depth was measured as the shortest distance between the liver capsule and the tumor surface on axial CT or MRI images. The CT value at the central area of tumor in the portal venous phase was also evaluated. The procedure-related data included the number of specimens obtained, type of imaging guidance (US only or CT-assisted with or without US), and the operator’s experience in performing PLTB.

### Final diagnoses

The final diagnoses were based on the pathological diagnoses or clinical follow-up with diagnostic imaging. When the pathological report was diagnostic for malignancy, the biopsy result was classified as “diagnostic”, and no additional follow-up was required. If the pathological result was diagnostic for a specific benign tumor, such as hemangioma, focal nodular hyperplasia, or angiomyolipoma, and follow-up confirmed the benignity of the lesion, the result was also considered as “diagnostic”. In cases when the result of the biopsy was indeterminate (e.g., inflammatory changes, fibrosis, or necrosis), the final diagnosis was established by pathological examination after re-biopsy or surgery, or clinical follow-up with diagnostic imaging. If malignancy was confirmed later, the result of the biopsy was classified as “non-diagnostic”. If the lesion subsequently disappeared or shrank in size, the lesion was considered as a non-neoplastic lesion and excluded from the risk factor analysis.

### Classification of non-diagnostic biopsy: technical error and targeting error

**Fig. 2:**
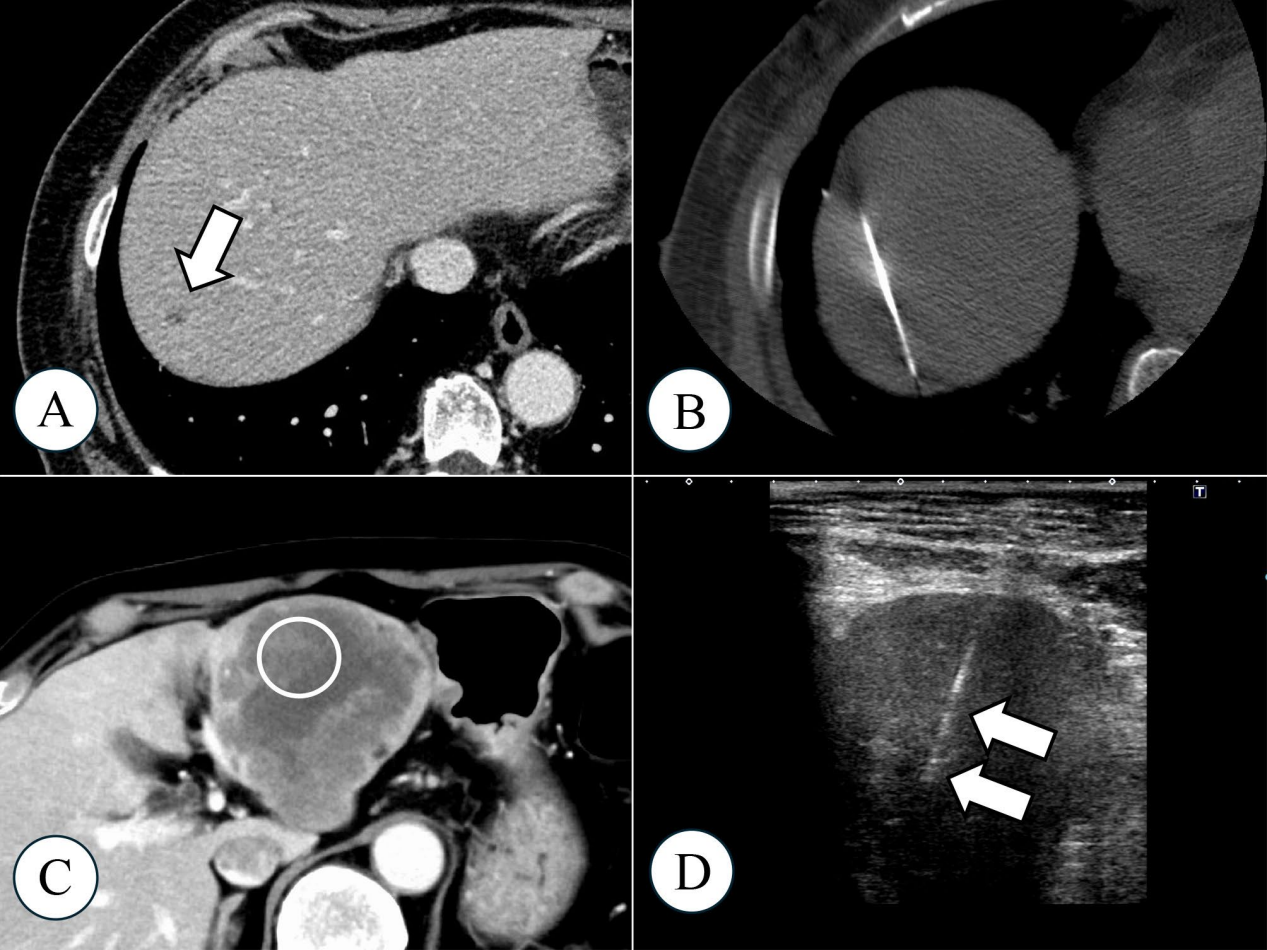
Classification of technical and targeting errors. **A** A tumor measuring 8 mm was detected in segment 7 on contrast-enhanced CT (arrow). **B** Percutaneous liver tumor biopsy was performed under CT guidance as the tumor was not detectable on ultrasound. Since the tumor was also undetectable on procedural CT imaging, tumor location was confirmed based on liver morphology and anatomy of the surrounding major vessels. The biopsy specimen was pathologically identified as liver tissue with fibrosis and did not contain any malignant cells. Partial hepatectomy was performed for this lesion, and evaluation of this specimen led to a diagnosis of hepatocellular carcinoma. This case was classified as a technical error. **C** A hypovascular tumor measuring 83 mm was confirmed in the lateral lobe on contrast-enhanced CT. The enhanced area in the tumor (circled area) was targeted. **D** The biopsy was performed under ultrasound guidance (arrow). The pathological diagnosis was necrotic tissue, and no malignant cells were found. Re-biopsy was performed from the peripheral zone of the tumor, and the pathological diagnosis confirmed adenocarcinoma (intrahepatic cholangiocarcinoma). This case was classified as a targeting error

The non-diagnostic biopsies were divided into two subgroups based on the cause of the error: the technical error and targeting error groups. A biopsy was classified as a technical error if the biopsy needle did not hit the target tumor (Fig. 2A, B). If the biopsy needle hit the target lesion but did not obtain a sufficient specimen for pathological diagnosis, it was classified as a targeting error (Fig. 2C, D). Two board-certified interventional radiologists (SK and TO; each with 9 years of experience as a radiologist) classified the biopsies based on preprocedural enhanced imaging, intraprocedural imaging, and pathological findings. The risk factors for non-diagnostic results were evaluated by comparing each of the two subgroups with the diagnostic group.

### Safety

Safety was evaluated using the Clavien–Dindo classification [22], and grade IIIa or higher events were designated as major complications.

### Statistical analysis

Categorical data are presented as numbers and percentages and continuous variables as medians and interquartile ranges (IQRs). The Fisher exact test or chi-squared test was used for the comparison of frequencies. The Student’s t-test or Mann–Whitney test was used to compare continuous variables, according to the distribution of data. Univariate analysis was performed to detect the factors affecting non-diagnostic results, where a p-value of 0.05 was considered significant and all tests were two-sided. Multivariate analysis was performed using a logistic regression model by incorporating pertinent variables with p-values < 0.05 in the univariate analysis. Receiver operating characteristic curves were generated to determine the cut-off value for continuous results to predict the risk factors for non-diagnostic results. The Youden index (maximum point of sensitivity + specificity − 1) was used to calculate the most appropriate cut-off value. All analyses were conducted using the Statistical Package for the Social Sciences version 27.0 (IBM, Armonk, NY, USA).

## Results

### Demographic characteristics, tumor-related, and procedural details

The participants' demographic characteristics, tumor-related, and procedural details are summarized in Table 1. The median maximum tumor size was 31 mm (IQR: 20–54), and 61% (573/938) of lesions were located in the right lobe. The median number of needle passes was 4 (IQR: 3–5); 92% (862/938) of biopsies were performed under US guidance only, and the remaining 8% (76/938) were performed under CT-assisted due to poor ultrasound visibility.

**Table 1:** Patient, tumor-related, and procedural characteristics

Variate	Value
Age, years	67 (54–71)
Sex	
Male, n (%)	445 (53)
Female, n (%)	398 (47)
BMI^a^	22 (20–25)
Tumor size, mm	31 (20–54)
Tumor location	
Right lobe, n (%)	573 (61)
Left lobe, n (%)	359 (38)
Caudate lobe, n (%)	6 (1)
Tumor depth, mm	9 (0–21)
CT value (portal venous phase), HU	65 (49–83)
Biopsy needle size	
18-G, n (%)	938 (100)
Fatty liver	134 (18)
Ascites	21 (2)
Number of specimens obtained	4 (3–5)
Imaging guidance	
US only, n (%)	862 (92)
CT-assisted, n (%)	76 (8)

Values are presented as the median (interquartile range) or number (percentage)

*BMI* body mass index, *HU* Hounsfield Unit

^a^Weight in kilograms divided by the square of height in meters

### Final diagnoses

**Fig. 3:**
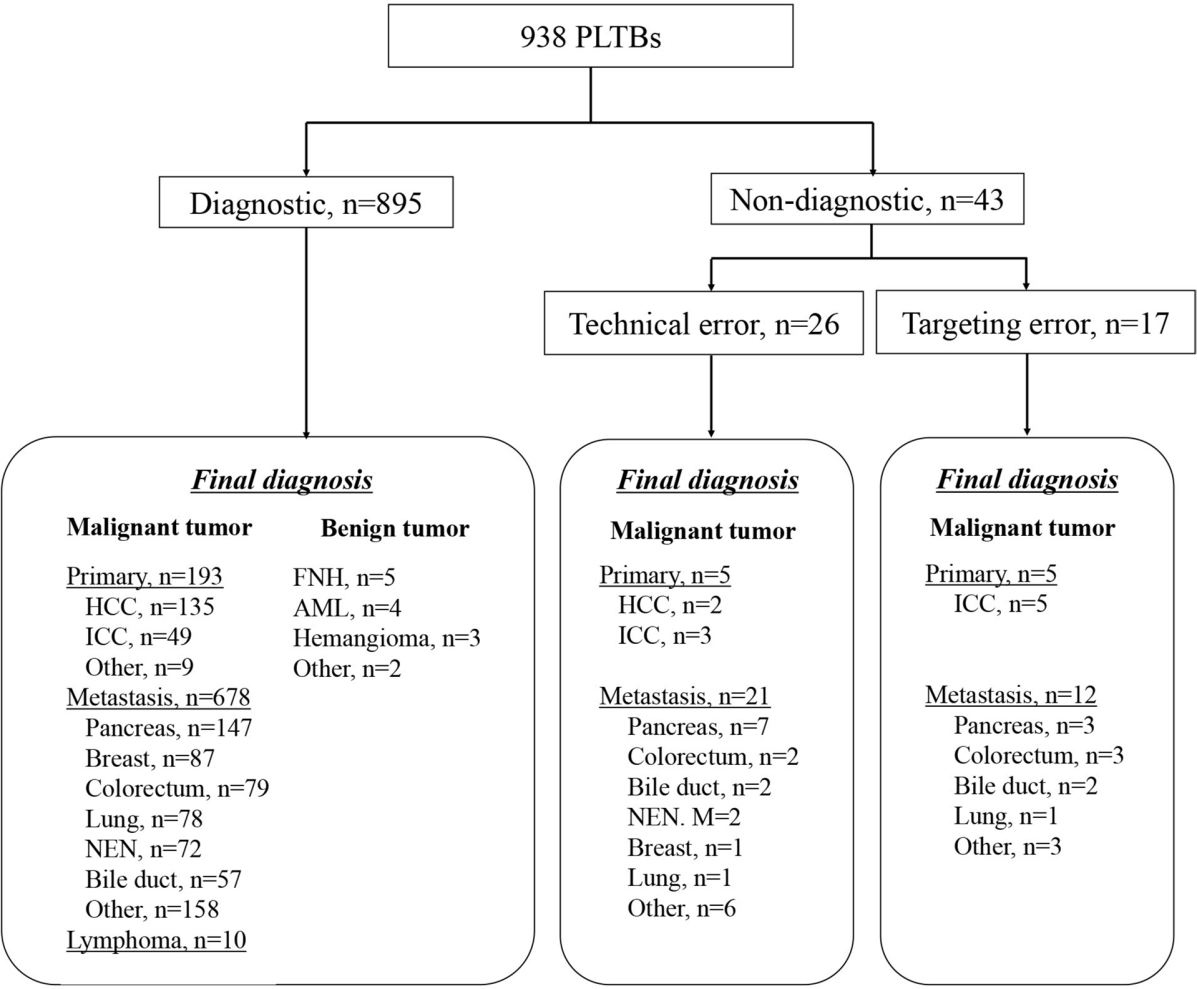
Flowchart of final diagnosis. *PLTB* percutaneous liver tumor biopsy, *HCC* hepatocellular carcinoma, *ICC* intrahepatic cholangiocarcinoma, *NEN* neuroendocrine neoplasm, *FNH* focal nodular hyperplasia, *AML* angiomyolipoma

881 and 14 biopsies were diagnosed as malignant tumor and specific benign tumor respectively, which were considered as “diagnostic biopsy”. The remaining 43 cases in which the biopsy result was indeterminate were diagnosed as malignant tumor depending on re-biopsy or surgery in 24 cases and clinical follow-up with diagnostic imaging after biopsy in 19 cases. These biopsies were classified as “non-diagnostic biopsy” (Fig. 3).

### Classification of non-diagnostic biopsies

The non-diagnostic rate was 4.6% (43/938). Of these, 26 biopsies were classified as technical error and 17 biopsies as targeting error. The differences between these two subgroups are summarized in Table 2. The median tumor size was significantly smaller in the technical error group than in the targeting error group (16 vs 59 mm, p < 0.001). The CT value was significantly lower in the targeting error group (p = 0.008), whereas the tumor depth was significantly greater in the technical error group (p = 0.032).

**Table 2:** Comparison of the technical error group and the targeting error group

Variate	Non-diagnostic biopsy (n = 43)	Subgroups		p value
		Technical error (n = 26)	Targeting error (n = 17)	
Age, years	63 (58–69)	63 (58–69)	63 (58–70)	0.921
Sex: male	23 (53)	14 (54)	9 (53)	0.954
BMI^a^	22 (20–25)	22 (20–26)	22 (21–24)	0.881
Tumor size, mm	31 (14–59)	16 (13–27)	59 (52–73)	< 0.001*
Tumor depth, mm	10 (0–20)	15 (5–33)	0 (0–17)	0.032*
Location: right lobe	23 (53)	13 (50)	10 (59)	0.571
Imaging guidance: CT-assisted	8 (19)	7 (27)	1 (6)	0.119
CT value, HU	57 (53–84)	64 (56–84)	52 (22–57)	0.008*
Number of specimens obtained	5 (3–5)	5 (3–5)	5 (3.5–7)	0.400
Fatty liver	10 (26)	7 (28)	3 (21)	0.721

Values are presented as the median (interquartile range) or number (percentage)

*BMI* body mass index, *HU* Hounsfield Unit

*p < 0.05

^a^Weight in kilograms divided by the square of height in meters

### Risk factors for technical error

**Fig. 4:**
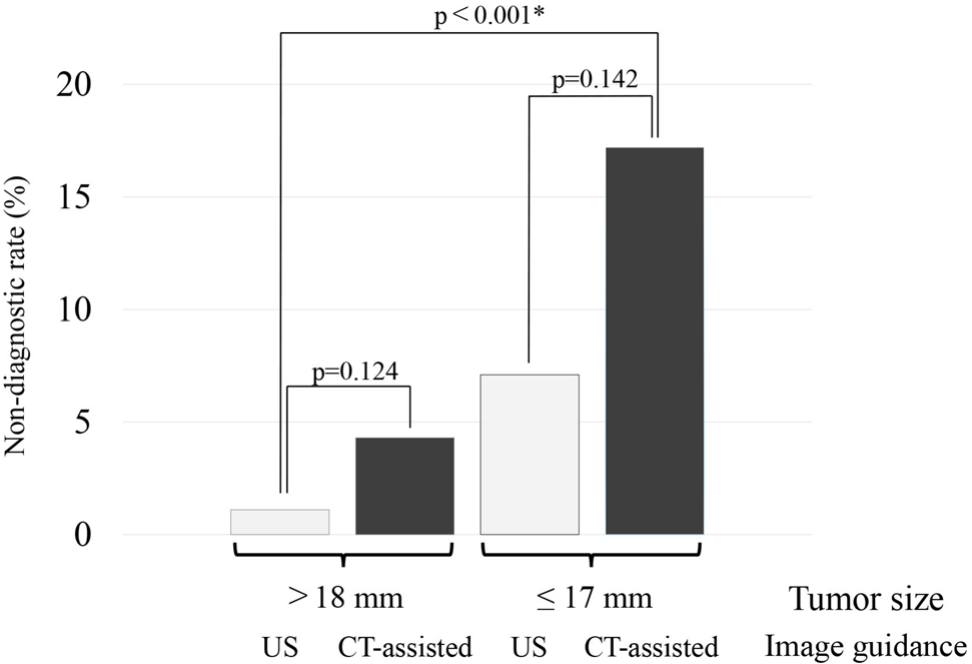
Non-diagnostic rate due to technical error based on the risk factors. Tumors with both risk factors (tumor size ≤ 17 mm and CT-assisted biopsy due to poor ultrasound visibility) had a significantly high risk of technical error compared to tumors without both factors (non-diagnostic rate: 17.2 vs 1.1%, p < 0.001). *p < 0.05

The cut-off value of tumor size was determined to be 17 mm, and the sensitivity, specificity, and area under the curve were 62, 81%, and 0.723 (95% CI 0.613–0.833), respectively. Multivariate analysis identified tumor diameter ≤ 17 mm and CT-assisted biopsy (p < 0.001 and p = 0.021, respectively) as significant risk factors for technical error (Table 3), and tumors with both factors had a significantly high risk of technical error compared to tumors without both factors (non-diagnostic rate: 17.2 vs 1.1%, p < 0.001) (Fig. 4).

**Table 3:** Univariate and multivariate analyses of factors affecting technical error

Variate		Univariate analysis			Multivariate analysis	
		Diagnostic	Technical error	p value	Odds ratio (95% CI)	p value
Age	≥ 65 years	409 (46)	12 (46)	0.963		
Sex	Male	483 (54)	14 (54)	0.990		
BMI^a^	≥ 30	36 (4)	1 (4)	1.000		
Tumor size	≤ 17 mm	166 (19)	16 (62)	< 0.001*	7.027 (2.687–13.952)	< 0.001*
Tumor depth	≥ 15 mm	337 (37)	13 (50)	0.204		
Location	Right lobe	548 (61)	15 (58)	0.715		
Imaging guidance	CT-assisted^c^	68 (8)	7 (27)	0.003*	3.032 (1.186–7.751)	0.021*
CT-value	≤ 55 HU	290 (36)	5 (22)	0.162		
Number of specimens obtained	≥ 5	336 (37)	13 (50)	0.200		
Operator’s experience^b^	≤ 50	382 (43)	14(54)	0.257		
Fatty liver	Yes	124 (18)	7 (28)	0.194		

Values are presented as number (percentage)

*p < 0.05

*BMI* body mass index, *HU* Hounsfield Unit, *CI* confidence interval

^a^Weight in kilograms divided by the square of height in meters

^b^The number of percutaneous liver tumor biopsies performed

^c^CT-assisted biopsy due to poor ultrasound visibility

### Risk factors for targeting error

The cut-off value of tumor size was determined to be 42 mm, and the sensitivity, specificity, and area under the curve were 94, 63%, and 0.779 (95% CI 0.713–0.845), respectively. Univariate analysis identified tumor diameter ≥ 42 mm and CT value ≤ 55 HU in the portal venous phase as risk factors for targeting error (p < 0.001 and p = 0.028, respectively). In the multivariate analysis, only tumor diameter ≥ 42 mm retained significance as a risk factor (p = 0.003) (Table 4).

**Table 4:** Univariate and multivariate analyses of factors affecting targeting error

Variate		Univariate analysis			Multivariate analysis	
		Diagnostic	Targeting error	p value	Odds ratio (95% CI)	p value
Age	≥ 65 years	409 (46)	7 (41)	0.711		
Sex	Male	483 (54)	9 (53)	0.933		
BMI^a^	≥ 30	36 (4)	0 (0)	1.000		
Tumor size	≤ 42 mm	329 (37)	16 (94)	< 0.001*	22.295 (2.922–170.139)	0.003*
Tumor depth	≥ 15 mm	337 (38)	4 (24)	0.231		
Location	Right lobe	549 (61)	10 (59)	0.825		
Imaging guidance	CT-assisted^c^	68 (8)	1 (6)	1.000		
CT-value	≤ 55 HU	290 (36)	10 (63)	0.028*	2.375 (0.844–6.689)	0.101
Number of specimens obtained	≥ 5	335 (37)	10 (6)	0.073		
Operator’s experience^b^	≤ 50	382 (43)	4 (24)	0.113		
Fatty liver	Yes	124 (18)	3 (22)	0.725		

Values are presented as number (percentage)

*p < 0.05

*BMI* body mass index, *HU* Hounsfield Unit, *CI* confidence interval

^a^Weight in kilograms divided by the square of height in meters

^b^The number of percutaneous liver tumor biopsies performed

^c^CT-assisted biopsy due to poor ultrasound visibility

### Safety

Major complications occurred in nine cases (9/938, 1.0%), all of which constituted intra-abdominal hemorrhage from the intrahepatic artery (eight cases) or intercostal artery (one case). Hemostasis was achieved by transarterial embolization in eight cases, and one patient underwent surgery owing to failure of transarterial embolization.

## Discussion

Multivariate analysis identified small lesion size (≤ 17 mm) and CT-assisted biopsy due to poor US visibility as significant risk factors for technical error, and large lesion size (≥ 42 mm) as a significant risk factor for targeting error.

In our study, the median tumor size of all non-diagnostic biopsies was the same as that of all PLTB cases. This was the result of offset by the characteristics of the technical and targeting error groups. The characteristics between the two groups were fundamentally different, especially regarding tumor size. It was suggested that the factors associated with non-diagnostic results should ideally be analyzed based on the cause of error, which may be able to identify the risk factors that are consistent with actual clinical questions. To our knowledge, the present study is the first to evaluate the risk factors based on the cause of error.

Technical error is a major cause of non-diagnostic biopsy. Some previous studies found an association between small tumor size and diagnostic failure [12, 20, 23]. Our results were consistent with these results. In addition, the present study determined the cut-off value (≤ 17 mm) based on receiver operating characteristic curve, which is useful information for preventing non-diagnostic results. Furthermore, alternative or complementary use of CT was determined as a risk factor for technical error. Whether to use CT or not depended on the visibility of the target tumor on US; namely, CT was used only for tumors with poor US visibility. CT-guidance was an option to identify the target lesion in such cases. However, CT-guided liver tumor biopsy has several problems [24]. First, the spatial resolution of CT is inferior to that of US. Second, intermittent CT-fluoroscopic guidance does not provide real-time imaging [25]. The tumor location is changed by respiratory movement. Therefore, it is difficult to confirm the exact location. Third, metal artifacts from needles degrade image quality. Thus, tumors with poor US visibility, such as small tumors or tumors located near the hepatic dome or hepatic hilum, are also difficult to identify using CT-assisted techniques. The use of iodine contrast media is one option that can improve the detectability of the target tumor. However, since the visualization provided by intravenous contrast media can be short-lived, the efficacy in improving the diagnostic accuracy of PLTB may be limited. To overcome this problem, administration of the contrast medium after insertion of the biopsy needle may be effective to confirm the location of the target tumor and the biopsy needle [26].

Targeting error is another cause of non-diagnostic results. A previous study investigating tumor size and diagnostic accuracy found that a diagnostic accuracy of 88.8% was achieved for tumors measuring 2–4 cm, which decreased not only for small tumors (< 2 cm: 80%) but also for large tumors (4–6 cm: 84.6%, ≥ 6 cm: 82.0%) [21]. This result is consistent with the results of the present study, and the deterioration of accuracy for large tumors is attributed to tumor necrosis or degeneration, which is more likely to occur in large tumors [27]. The present study determined the cut-off value (≥ 42 mm) as a risk factor for targeting error. For these large tumors with heterogeneity, the puncture site must be carefully evaluated based on preoperative or intraoperative imaging.

This study determined the cut-off value of tumor size based on statistical analysis of a large number of cases. Our findings are clinically valuable because several measures can be implemented in advance to avoid non-diagnostic results in cases of PLTB with a high risk of non-diagnostic results. For example, the efficacy of contrast agents for US has been reported [12, 21, 28, 29]. These agents can improve tumor detectability and clarify the difference between viable and necrotic areas, potentially contributing to the prevention of non-diagnostic results. Besides, rapid on-site cytologic evaluation has been reported to improve diagnostic accuracy [20, 30]. Ideally, these techniques should be utilized routinely to improve diagnostic accuracy. However, in situations where it is difficult to use these techniques for all PLTBs, one option may be to use them only in cases with a high risk of non-diagnostic results, as shown herein. In addition, an experienced operator can oversee PLTB for tumors at high risk for non-diagnostic biopsy. These considerations may be beneficial for patients by avoiding unnecessary invasion and delay caused by non-diagnostic results.

The present study had several limitations. First, its retrospective design and single-center setting may limit the generalizability of the findings. However, the procedural uniformity and inclusion of a large number of cases minimized this effect. Second, PLTB was performed by multiple interventional radiologists. The diagnostic accuracy of PLTB may have been affected by selection of the target lesion from multiple lesions, selection of the target area in a heterogeneous lesion, and choice of imaging modality for guidance left to the individual radiologist’s discretion. Third, PLTB was performed without contrast-enhanced US or rapid on-site cytologic evaluation. Our results may not be extrapolated to PLTB using these techniques.

In conclusion, tumor size ≤ 17 mm and CT-assisted biopsy due to poor US visibility are risk factors for technical error, and tumor size ≥ 42 mm is a risk factor for targeting error in PLTB. For these tumors, implementing special measures in advance may be effective at avoiding non-diagnostic results.
